# Japanese Encephalitis Surveillance and Immunization — Asia and the Western Pacific, 2012

**Published:** 2013-08-23

**Authors:** Samir Baig, Kimberley K. Fox, Youngmee Jee, Patrick O’Connor, Joachim Hombach, Susan A. Wang, Terri Hyde, Marc Fischer, Susan L. Hills

**Affiliations:** Regional Office for the Western Pacific; Regional Office for South-East Asia; World Health Organization; Global Immunizations Div, Center for Global Health; Div of Vector-Borne Diseases, National Center for Emerging and Zoonotic Infectious Diseases, CDC

Japanese encephalitis (JE) virus is a leading cause of encephalitis in Asia, causing an estimated 67,900 JE cases annually. To control JE, the World Health Organization (WHO) recommends that JE vaccine be incorporated into immunization programs in all areas where JE is a public health problem. For many decades, progress mainly occurred in a small number of high-income Asian countries. Recently, prospects for control have improved with better disease burden awareness as a result of increased JE surveillance and wider availability of safe, effective vaccines. This report summarizes the status of JE surveillance and immunization programs in 2012 in Asia and the Western Pacific. Data were obtained from the WHO/United Nations Children’s Fund (UNICEF) Joint Reporting Form (JRF), published literature, meeting reports, and websites. In 2012, 18 (75%) of the 24 countries with areas of JE virus transmission risk conducted at least some JE surveillance, and 11 (46%) had a JE immunization program. Further progress toward JE control requires increased awareness of disease burden at the national and regional levels, availability of WHO-prequalified pediatric JE vaccines, and international support for surveillance and vaccine introduction in countries with limited resources.

JE is a mosquito-borne disease with a 20%–30% case-fatality rate and neurologic or psychiatric sequelae in 30%–50% of survivors (1). JE virus is transmitted in an enzootic cycle between mosquitoes and amplifying vertebrate hosts, primarily pigs and wading birds. Humans are incidental hosts in the JE virus transmission cycle. In endemic regions, JE occurs mainly among children aged ≤15, years and risk is highest in rural, agricultural areas. No specific treatment for JE is available. Although the use of insecticides and improvements in agricultural practices (e.g., centralized pig production) might contribute to reduction of disease incidence, vaccination is the single most important preventive measure (2). Twenty-four WHO member states have areas of JE virus transmission risk (Figure) (1). Risk areas are determined based on any evidence of JE virus transmission from human surveillance, mosquito or animal studies, or ecologic similarity to areas with proven transmission.

Information on JE surveillance and immunization programs was obtained from JRF reports for 2012, from JRF reports for 2011 of reported cases (because 2012 data were incomplete) (3), reports from the Fifth Biregional Meeting on JE Prevention and Control and the Third JE Laboratory Network Meeting in the Western Pacific Region in 2011 (4,5), Ministry of Health websites, and published English-language literature. Information collected on JE surveillance programs included strategies, age groups, diagnostic testing availability, case numbers reported on the JRF, and use of the WHO case definition for acute encephalitis syndrome (AES) to identify suspected cases. WHO defines AES as the acute onset of fever and either altered mental status, new onset of seizures, or both; an AES case in which laboratory testing confirms acute JE virus infection is considered a JE case (6). For JE immunization programs, data collected included program strategies, ages for routine vaccination, and vaccines used.

## Surveillance programs

In 2012, 18 (75%) of the 24 countries with JE virus transmission risk conducted JE surveillance (Table 1). Five (21%) of the 24 countries conducted surveillance nationally or in appropriate geographic risk areas and routinely tested suspected cases for JE, six (25%) conducted national surveillance without laboratory confirmation of every suspected case, and seven (29%) conducted surveillance at sentinel sites. Countries with sentinel surveillance had a median of five surveillance sites (range: 2–125), and laboratory testing usually was available for suspected cases. Surveillance was conducted among all age groups in 16 (89%) of the 18 countries and only in children in two countries. The WHO AES case definition was used to identify suspected cases in seven countries.* The remaining 11 countries either used a locally developed surveillance case definition for encephalitis or meningitis/encephalitis, or clinicians were asked to investigate and report clinically compatible cases.

In 2011, 19 (79%) of the 24 countries reported a total of 10,426 JE cases. The 8,247 reported cases from India and 1,625 from China represented 95% of all cases; no other country reported >150 cases. Among five countries reporting zero cases, three had no surveillance program.

## Immunization Programs

Eleven (46%) of the 24 countries with JE virus transmission risk had a JE immunization program in 2012. Seven (29%) programs were implemented nationally or in all areas considered to have JE risk, and four (17%) were subnational and did not include all risk areas (Table 2). Ten (42%) countries included JE vaccine in the routine vaccination schedule, and one country conducted annual vaccination campaigns. The scheduled age for beginning vaccination ranged from 8 months to 3 years. Vaccines used included inactivated mouse brain–derived vaccines (five countries), live attenuated SA 14-14-2 vaccine (five countries), and inactivated Vero cell culture-derived vaccines (two countries).†

### Editorial Note

Substantial progress has been made in JE surveillance and immunization programs since the 1990s. In 2012, three quarters of countries with JE virus transmission risk conducted JE surveillance and almost half had a JE immunization program. Previously, data were often only collected for short-term studies, and JE prevention activities were limited to a few countries (7). Recent progress has been spurred by an increase in funding, availability of improved vaccines, and growing international attention to the disease.

JE surveillance has improved in many countries in terms of data quality, numbers of surveillance sites, integration into national health systems, and use of a standard case definition (8). During the past 2 years, government surveillance programs have been newly established in the Philippines, Bhutan, and Papua New Guinea. WHO has developed a JE laboratory network that provides training workshops, technical support, proficiency testing, and confirmatory testing for 18 countries. Improved surveillance with better understanding of the burden and geographic extent of disease has resulted in greater recognition of JE as a public health problem in some countries (e.g., Laos), availability of data to support decision making on vaccine introduction (e.g., Cambodia), and better data to monitor the effectiveness of immunization programs and guide program expansion or improvement (e.g., Nepal).

What is already known on this topic?

Japanese encephalitis (JE) virus is a leading cause of encephalitis in Asia. The World Health Organization recommends that JE vaccine be incorporated into immunization programs in all areas where JE is a public health problem. For many decades, progress with JE control mainly occurred in a small number of high-income Asian countries.

What is added by this report?

A review of surveillance and immunization program data in the 24 countries with JE virus transmission risk showed that in 2012, 18 of the countries performed at least some surveillance for JE, and 11 had a JE immunization program. This represents substantial progress, but many challenges remain.

What are the implications for public health practice?

To support further progress toward JE control, measures to maintain and improve awareness of JE disease burden, technical and financial support from international agencies and donors, and sustained national commitment are required.

Despite such progress, some countries still have limited data, and many challenges remain. JE case reporting is known to be incomplete and inaccurate. A recent review estimated that approximately 67,900 JE cases typically occur annually in this region, but in 2011, only 10,426 were reported (1). Although the case numbers reported on the JRF likely include some non-JE cases (AES cases without laboratory confirmation), underreporting of JE because of the limited scope of surveillance is a more important issue. Although sentinel surveillance is recommended where nationwide surveillance would be logistically complex or financially burdensome, it does not provide population-based disease burden estimates, and findings reflect disease patterns at sites that might be selected for operational rather than epidemiologic reasons. Incomplete laboratory confirmation of cases can occur when sample collection is inadequate or access to testing is limited. JE case counts are not comparable across countries because of varying use of laboratory confirmation. In addition, AES case numbers cannot be compared because of variability in clinical definitions used to identify cases, with the WHO AES case definition not used in the majority of countries that conduct surveillance.

WHO recommends that JE vaccination be extended to all areas where JE is a public health problem (2). The most effective immunization strategy is a one-time campaign in the locally defined target population (i.e., those age groups determined to be most at risk), followed by incorporation of JE vaccine into the routine childhood immunization program (2). Insufficient disease burden data, financial constraints, and competing vaccine priorities have prevented some countries from implementing programs. Several countries (e.g., India, Nepal, Sri Lanka, Thailand, and Vietnam) have initially targeted higher-risk areas and later expanded their programs. Countries with limited JE virus transmission risk might not have sufficient disease burden to require a JE vaccination program.

Three types of JE vaccine are used in national immunization programs. Inactivated mouse brain–derived JE vaccine has been available for >50 years, but has a multidose primary and booster schedule and is relatively expensive; its use has declined recently. Live attenuated SA14-14-2 vaccine has a simple schedule and good safety profile, and improved international availability has led to increased use in recent years. The vaccine’s manufacturer, Chengdu Institute of Biological Products, has guaranteed a maximum public sector price for lower-income countries, comparable to the international public sector price for measles vaccine (9). Several inactivated Vero cell culture-derived vaccines also have become available and are being used in national programs. Despite the availability of pediatric clinical trial data and extensive post-licensure experience for several vaccines, until recently, no JE vaccine manufacturer had made a submission to WHO for prequalification of a JE vaccine, a process that reviews a vaccine’s quality, safety, efficacy, and programmatic suitability. However, at least one JE vaccine is currently under review for pediatric use. WHO prequalification will enable procurement by United Nations agencies. The GAVI Alliance also has indicated it will consider funding JE vaccination when a WHO-prequalified vaccine for children is available (10).

The findings in this report are subject to at least one limitation. Data for this report were collected from publicly available sources with varying levels of detail and might not reflect the most current information.

Immunization is the most effective JE prevention strategy and has been shown in studies in several countries to be cost-effective (8). Recently, the Technical Advisory Group on Immunization and Vaccine-Preventable Diseases in the Western Pacific Region endorsed development of an accelerated control goal for JE. Despite progress with surveillance and immunization, challenges remain. Measures to improve awareness of JE disease burden, technical and financial support from international agencies and donors, and sustained national commitment are required to support progress toward improved JE control.

## Figures and Tables

**FIGURE:**
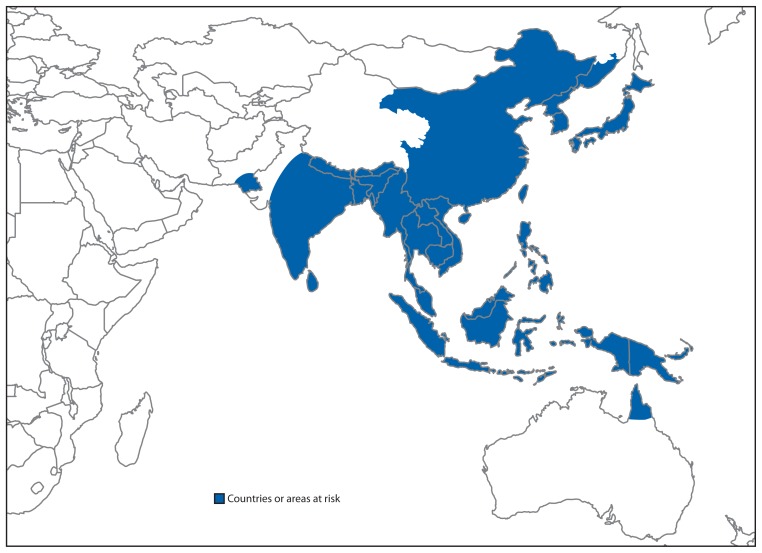
Geographic distribution of Japanese encephalitis, 2012

**TABLE 1:** Characteristics of Japanese encephalitis (JE) surveillance in countries with JE virus transmission risk, 2012

Country	JE surveillance program	Age groups under surveillance	Laboratory confirmation of cases
Australia*	Risk areas†	All	Yes
Bangladesh	Sentinel (2 sites)	All	Yes
Bhutan	Sentinel (5 sites)	All	Yes
Brunei Darussalam	None	—	—
Burma (Myanmar)	National	All	Partial
Cambodia	Sentinel (6 sites)	≤15 yrs	Yes
China	National§	All	Partial
Taiwan	All areas	All	Yes
India	Sentinel (~50 sites)	All	Partial
Indonesia	None	—	—
Japan	National	All	Yes
Laos	National	All	Partial
Malaysia	National	All	Partial
Nepal	Sentinel (125 sites)¶	All	Yes
North Korea	None	—	—
Pakistan	None	—	—
Papua New Guinea	Sentinel (2 sites)	≤12 yrs	Yes
Philippines	Sentinel (3 sites)	All	Yes
Russia*	None	—	—
Singapore	National	All	Yes
South Korea	National	All	Yes
Sri Lanka	National	All	Partial
Thailand	National	All	Yes
Timor-Leste	None**	—	—
Vietnam	National§	All	Partial

* JE virus transmission risk in well-defined, limited areas.

† Torres Strait Islands and northern Cape York.

§ Includes specified sentinel sites (24 in China and six in Vietnam) for comprehensive case-based surveillance with laboratory testing of every case.

¶ Sentinel sites located in every district in the country.

** Surveillance was implemented in 2009 but has not been maintained.

**TABLE 2:** Characteristics of Japanese encephalitis (JE) immunization programs in countries with JE virus transmission risk, 2012

Country	JE immunization program	Strategy	Scheduled age to begin routine immunization	Vaccine used
Australia*	Targeted risk areas†	Routine	12 mos	MB§
Bangladesh	None	—	—	—
Bhutan	None	—	—	—
Brunei Darussalam	None	—	—	—
Burma (Myanmar)	None	—	—	—
Cambodia	Subnational¶	Routine	10 mos	LAV
China	National**	Routine	8 mos	LAV, VC
Taiwan	All areas	Routine	15 mos	MB
India	Risk areas††	Routine	16–24 mos	LAV
Indonesia	None	—	—	—
Japan	National	Routine	36 mos	VC
Laos	None	—	—	—
Malaysia	Subnational	Routine and outbreak response¶¶	9 mos	MB
Nepal	Subnational***	Routine	12 mos	LAV
North Korea	N/A§§	N/A	N/A	N/A
Pakistan	None	—	—	—
Papua New Guinea	None	—	—	—
Philippines	None	—	—	—
Russia*	None	—	—	—
Singapore	None	—	—	—
South Korea	National	Routine	12–24 mos	MB
Sri Lanka	National	Routine	9 mos	LAV
Thailand	National	Routine	18 mos	MB
Timor-Leste	None	—	—	—
Vietnam	Subnational†††	Annual campaign	12 mos	MB

**Abbreviations:** N/A = no information available; LAV = live attenuated SA 14-14-2 JE vaccine; VC = inactivated Vero cell culture-derived JE vaccine; MB = inactivated, mouse brain–derived JE vaccine.

* JE virus transmission risk in well-defined, limited areas.

† Vaccination recommended for residents of the outer Torres Strait Islands or nonresidents living or working there for ≥30 days during the wet season.

§ Program was temporarily suspended when MB became unavailable in 2010 and will recommence when a new pediatric JE vaccine is available.

¶ Three provinces.

** Excluding the nonendemic provinces of Qinghai, Tibet, and Xinjiang.

†† Routine program implemented in districts that have conducted campaigns; campaigns conducted in 109 endemic districts in 15 states during 2006–2011, and repeated in nine districts in two states in 2010.

§§ Mass campaign was conducted in 2010.

¶¶ In Sarawak, vaccination is provided as part of the routine childhood immunization program; in peninsular Malaysia and Sabah, vaccination is provided to children aged <15 years in the vicinity of an outbreak.

*** Routine program implemented in the 31 districts that have conducted campaigns.

††† Program commenced in high-risk districts in 1997 and reached approximately 80% of all districts in 2012.

## Footnotes

* Bhutan, India, Laos, Burma (Myanmar), Nepal, Papua New Guinea, and the Philippines.

† Live attenuated SA 14-14-2 vaccine and inactivated Vero cell culture-derived vaccine were used on mainland China, and inactivated mouse brain–derived vaccine was used on Taiwan.

## References

[1] Campbell GL, Hills SL, Fischer M (2011). Estimated global incidence of Japanese encephalitis: a systematic review. Bull World Health Organ.

[2] World Health Organization (2006). Japanese encephalitis vaccines. Wkly Epidemiol Rec.

[3] World Health Organization (2013). WHO/UNICEF joint reporting process.

[4] World Health Organization (2013). Meeting report. Fifth Biregional Meeting on Japanese Encephalitis Prevention and Control.

[5] World Health Organization (2013). Meeting report. Third Japanese Encephalitis Laboratory Network Meeting in the Western Pacific Region.

[6] World Health Organization (2008). WHO-recommended standards for surveillance of selected vaccine-preventable diseases.

[7] Tsai TF (2000). New initiatives for the control of Japanese encephalitis by vaccination: minutes of a WHO/CVI meeting, Bangkok, Thailand, 13–15 October 1998. Vaccine.

[8] Fischer M, Hills S, Staples E, Scheld WM, Hammer SM, Hughes JM (2008). Japanese encephalitis prevention and control: advances, challenges, and new initiatives. Emerging Infections 8.

[9] Yaïch M (2009). Investing in vaccines for developing countries: how public-private partnerships can confront neglected diseases. Hum Vaccine.

[10] GAVI Alliance (2008). GAVI vaccine investment strategy—Japanese encephalitis analysis.

